# Supplementary material for: Establishing Height-for-Age Z-Score Growth Reference Curves and Stunting Prevalence in Children and Adolescents in Pakistan
Source: Int J Environ Res Public Health. 2022 Oct 3;19(19):12630. doi: 10.3390/ijerph191912630 (PMC9566739; doi:10.3390/ijerph191912630)
Supplement: Supplementary file 1 [file ijerph-19-12630-s001.zip › ijerph-1895453-supplementary.pdf]

Table S1. Age-and- gender-specific smoothed height (cm) percentiles for the Pakistani children and adolescents, aged 2-18 years.

Age (years)	Percentiles								
	3rd	5th	10th	25th	50th	75th	90th	95th	97th
**Boys**									
2	86.39	87.89	89.98	93.04	95.95	98.50	100.56	101.70	102.41
3	91.48	93.03	95.35	99.05	102.92	106.59	109.74	111.56	112.72
4	96.75	98.20	100.47	104.37	108.81	113.41	117.68	120.29	122.01
5	101.61	102.99	105.19	109.12	113.88	119.12	124.35	127.72	130.02
6	106.03	107.37	109.53	113.46	118.36	123.94	129.72	133.57	136.27
7	110.14	111.48	113.64	117.58	122.51	128.14	134.00	137.92	140.68
8	114.12	115.49	117.70	121.68	126.57	132.07	137.66	141.34	143.89
9	118.14	119.58	121.87	125.93	130.80	136.10	141.30	144.63	146.88
10	122.29	123.84	126.29	130.52	135.44	140.60	145.48	148.50	150.51
11	126.54	128.28	130.98	135.53	140.64	145.81	150.53	153.36	155.22
12	130.74	132.74	135.78	140.78	146.18	151.46	156.13	158.87	160.64
13	134.88	137.16	140.56	146.01	151.73	157.16	161.84	164.54	166.26
14	139.12	141.60	145.26	151.02	156.97	162.51	167.23	169.92	171.64
15	143.77	146.23	149.87	155.62	161.56	167.12	171.85	174.57	176.29
16	148.89	151.06	154.31	159.59	165.19	170.57	175.25	177.97	179.71
17	154.02	155.70	158.30	162.67	167.53	172.41	176.84	179.49	181.22
18	158.37	159.53	161.36	164.54	168.25	172.18	175.92	178.26	179.82
**Girls**									
2	88.81	89.54	90.73	92.97	95.91	99.56	103.75	106.87	109.25
3	92.30	93.25	94.82	97.71	101.41	105.81	110.59	113.94	116.37
4	95.85	97.01	98.90	102.32	106.59	111.47	116.53	119.92	122.30
5	99.68	100.99	103.10	106.88	111.51	116.67	121.88	125.27	127.61
6	103.86	105.25	107.47	111.43	116.23	121.53	126.80	130.20	132.52
7	108.33	109.73	111.98	115.98	120.82	126.13	131.40	134.79	137.10
8	112.91	114.31	116.54	120.52	125.32	130.58	135.80	139.16	141.44
9	117.43	118.83	121.06	125.03	129.79	135.01	140.14	143.44	145.67
10	121.69	123.13	125.43	129.48	134.31	139.52	144.60	147.82	149.99
11	125.48	127.05	129.52	133.83	138.87	144.20	149.27	152.43	154.54
12	128.71	130.48	133.24	137.96	143.33	148.84	153.94	157.04	159.08
13	131.40	133.43	136.54	141.74	147.47	153.17	158.29	161.34	163.32
14	133.73	136.01	139.45	145.08	151.10	156.93	162.04	165.02	166.94
15	136.08	138.49	142.09	147.89	154.01	159.85	164.89	167.81	169.68
16	138.87	141.15	144.58	150.13	156.01	161.64	166.54	169.39	171.21
17	142.11	144.00	146.88	151.65	156.88	162.03	166.63	169.34	171.10
18	145.23	146.58	148.69	152.30	156.43	160.68	164.64	167.06	168.65
